# Association between migraine, lifestyle and socioeconomic factors: a population-based cross-sectional study

**DOI:** 10.1007/s10194-011-0321-9

**Published:** 2011-03-10

**Authors:** Han Le, Peer Tfelt-Hansen, Axel Skytthe, Kirsten Ohm Kyvik, Jes Olesen

**Affiliations:** 1 Department of Neurology, The Danish Headache Centre, Glostrup Hospital, Faculty of Health Sciences, University of Copenhagen, 2600 Glostrup, Denmark; 2 The Danish Twin Registry, Epidemiology, Institute of Public Health, University of Southern Denmark, Odense, Denmark; 3 Institute of Regional Health Services Research, University of Southern Denmark, Odense, Denmark

**Keywords:** Migraine, Socioeconomic factors, Smoking, Alcohol, Body mass index

## Abstract

To investigate whether sex-specific associations exist between migraine, lifestyle or socioeconomic factors. We distinguished between the subtypes migraine with aura (MA) and migraine without aura (MO). In 2002, a questionnaire containing validated questions to diagnose migraine and questions on lifestyle and socioeconomic factors was sent to 46,418 twin individuals residing in Denmark. 31,865 twin individuals aged 20–71 were included. The twins are representative of the Danish population with regard to migraine and other somatic diseases and were used as such in the present study. An increased risk of migraine was significantly associated with lower level of schooling and education, retirement, unemployment, and smoking. A decreased risk of migraine was significantly associated with heavy physical exercise and intake of alcohol. Direct comparison between the subtypes showed a decreased risk of MA compared to MO in subjects with low education or weekly intake of alcohol. The risk of MA was increased compared to MO in unemployed or retired subjects. Direct comparison between sexes showed a decreased risk of migraine for men compared to women in subjects who were low educated, unemployed or studying. The risk was increased for men compared to women in subjects with heavy physical exercise, intake of alcohol, and body mass index >25. Migraine was associated with several lifestyle and socioeconomic factors. Most associations such as low education and employment status were probably due to the negative effects of having migraine while others such as smoking were risk factors for migraine.

## Introduction

Several twin studies have shown that the heritability of the migraine phenotype is approximately 34–65% [1–3]. The rest should, therefore, be accounted for by environmental factors. Some factors may affect migraine in a positive or negative way and migraine may influence on lifestyle or socioeconomic status. It is intuitively obvious that having many attacks of severe pain, nausea and other symptoms, often binding the sufferer to bed for 1 or 2 days, may adversely affect their functioning and, thus, may lead to less social advancement.

Some large-scale studies have found migraine to be associated with low socioeconomic status [4, 5] and some have not [6, 7]. The association with factors such as tobacco, alcohol, and body mass index (BMI) still needs further investigation in large population-based studies. Most studies have generally not distinguished between migraine with aura (MA) and migraine without aura (MO) but they are distinct entities [8–11] and are likely to have different associations.

The aim of the present study was to analyse the association between migraine and a number of lifestyle and socioeconomic factors and to discuss for each factor whether a difference between migraine subjects and healthy subjects is most likely a risk factor for disease development or a consequence of having migraine.

The Danish “Twin Omnibus 2002” was a questionnaire study among 46,000 twin individuals, which included questions on migraine and lifestyle and socioeconomic factors [12]. These twins are representative of the whole Danish population with regard to migraine [13] as well as several other diseases, e.g., diabetes [14], asthma [15] and mortality [16]. In the present publication, the twin status is disregarded and the material is regarded as a random sample from the Danish population. Our very large sample size has the statistical power to not only detect potential factors linked to migraine, in general, but also to reveal differences between men and women and between cases with MA and MO.

## Methods

The Danish Twin Registry (DTR) is the oldest and most complete national twin registry in the world [17]. The ascertainment of twins has been carried out in a variety of ways, but for the twins enrolled in this study the Danish Civil Registration System which has registered all persons living in Denmark since 1 April 1968 was used [12, 17, 18]. The procedure for identifying twins born between 1931–1952 was based on an algorithm extracting all persons born on the same date, in the same parish and with the same historical surname [17]. Twins born between 1953–1982 were identified via the mother assuming that a woman giving birth to more than one child within three consecutive days had twins [19]. The DTR is validated and is representative of the Danish population with regard to migraine [13].

The “Twin Omnibus 2002” was a questionnaire sent to 46,418 Danish twin individuals aged 20–71 and 34,944 (75%) responded. Of these 31,865 had answered both migraine questions and were included while the rest were excluded due to missing data. Questions included self-reported migraine and several lifestyle and socioeconomic factors such as: school level, educational level, employment status, marital status, physical work load, recreational physical activity, smoking status, alcohol consumption, weight, height, and self-rated health by short form (SF)-12. BMI was calculated from the height and weight measurements and categorized according to the World Health Organization’s BMI classification. Self-reported migraine cases were classified and further subdivided into self-reported MA and MO based on the following screening questions:

1. Have you ever had migraine?
2. Have you ever had visual disturbances that lasted from 5 to 60 min and were succeeded by a headache?

Validation of the screening questions showed that it is possible to identify 76% of all subjects with migraine, 85% of all with MA and 72% of all with MO. The number of self-reported migraine, MA, and MO cases was 8,044, 3,086, and 4,958, respectively, and there were 23,821 subjects without migraine, for the purpose of the present study called healthy subjects. There were more women (69.6%) than men (30.4%) in the migraine group whereas no significant difference was found in the group without migraine. The median age differed with 1 year between the migraine group (women = 43; men = 45) and the group without migraine (women = 42; men = 44). A detailed description of the diagnostic criteria for migraine cases, validation of screening questions and demographic factors is being published elsewhere [20].

### Data analyses

Univariate analyses were used to investigate the association between migraine and all the determinants except SF-12. Statistical significance was tested with χ^2^ test. *p* values < 0.05 were considered significant. We subsequently conducted multivariate analyses by means of logistic regression to control for confounding. Each determinant was adjusted for all the other determinants and age except “school level” where “educational level” was not included in the logistic regression model, because this was a factor in the causal pathway. In men, there was a negligible number (*n* < 5) in the category called homemaker in “employment status”. Estimations made for men did not include this category in the logistic regression model. The results were given as odds ratios (OR) with 95% confidence intervals (CI). Independent samples *t* test was conducted to compare the SF-12 scores of migraine subjects and healthy subjects. All analyses were stratified by sex and migraine subtypes. Data analyses were performed with SPSS version 18.0.

## Results

The distribution of self-reported migraine, MA and MO by lifestyle and socioeconomic factors are presented for all subjects in Table 1 and for women and men in Table 2. The crude and adjusted risk of migraine, MA and MO by lifestyle and socioeconomic factors are presented for all subjects in Table 3 and for women and men in Table 4.

**Table 1:** The distribution of self-reported migraine, MA and MO by lifestyle and socioeconomic factors in all subjects

	Migraine *n* (%)	MA *n* (%)	MO *n* (%)	Healthy *n* (%)
School level	*p* < 0.001	*p* = 0.002	*p* < 0.001	–
Graduated 12th grade	2,504 (32.2)	999 (33.5)	1,505 (31.5)	8,147 (35.4)
Graduated 9th grade	3,651 (47.0)	1,413 (47.3)	2,238 (46.8)	10,117 (43.9)
Not graduated 9th grade	1,616 (20.8)	574 (19.2)	1,042 (21.8)	4,766 (20.7)
Educational level	*p* < 0.001	*p* = 0.001	*p* < 0.001	–
Higher education >4 years	543 (7.5)	242 (8.7)	301 (6.8)	2,280 (10.7)
Higher education ≤4 years	2,230 (30.9)	861 (31.1)	1,369 (30.8)	6,033 (28.4)
Vocational education	3,183 (44.1)	1,183 (42.8)	2,000 (45.0)	9,346 (44.0)
No education	1,258 (17.4)	481 (17.4)	777 (17.5)	3,594 (16.9)
Employment status	*p* < 0.001	*p* < 0.001	*p* < 0.001	–
Employed	4,820 (69.6)	1,815 (67.7)	3,005 (70.7)	14,468 (69.3)
Self-employed	491 (7.1)	173 (6.5)	318 (7.5)	1,853 (8.9)
Homemaker	268 (3.9)	99 (3.7)	169 (4.0)	577 (2.8)
Retired	621 (9.0)	277 (10.3)	344 (8.1)	1,514 (7.3)
Student	544 (7.9)	226 (8.4)	318 (7.5)	2,061 (9.9)
Unemployed	185 (2.7)	89 (3.3)	96 (2.3)	407 (1.9)
Physical work load	*p* < 0.001	*p* < 0.001	*p* < 0.001	–
Mainly seated	1,881 (25.0)	740 (25.6)	1,141 (24.6)	6,429 (28.7)
Seated/standing or walking	5,060 (67.3)	1,947 (67.4)	3,113 (67.1)	14,134 (63.0)
Heavy physical work	583 (7.7)	200 (6.9)	383 (8.3)	1,876 (8.4)
Recreational physical activity	*p* < 0.001	*p* < 0.001	*p* < 0.001	–
No physical exercise	1,416 (18.6)	536 (18.3)	880 (18.7)	3,803 (16.7)
Light physical exercise	4,870 (63.9)	1,882 (64.4)	2,988 (63.6)	14,056 (61.7)
Heavy physical exercise	1,335 (17.5)	506 (17.3)	829 (17.6)	4,912 (21.6)
BMI	*p* < 0.001	*p* < 0.001	*p* < 0.001	–
Underweight	230 (3.0)	91 (3.1)	139 (3.0)	477 (2.1)
Normal weight	4,621 (60.8)	1,809 (62.1)	2,812 (59.9)	13,430 (59.4)
Overweight	2,072 (27.2)	762 (26.2)	1,310 (27.9)	6,973 (30.8)
Obese class I–III	682 (9.0)	249 (8.6)	433 (9.2)	1,743 (7.7)
Smoking status	*p* < 0.001	*p* = 0.003	*p* = 0.006	–
Non-smoker	3,270 (41.8)	1,242 (41.3)	2,028 (42.1)	10,346 (44.4)
Former smoker	1,762 (22.5)	679 (22.6)	1,083 (22.5)	5,136 (22.1)
Current smoker	2,795 (35.7)	1,085 (36.1)	1,710 (35.5)	7,794 (33.5)
Alcohol consumption	*p* < 0.001	*p* < 0.001	*p* < 0.001	–
Never/seldom	2,200 (27.7)	911 (29.8)	1,289 (26.3)	4,431 (18.9)
Monthly	1,735 (21.8)	640 (21.0)	1,095 (22.4)	4,827 (20.5)
Weekly	4,009 (50.5)	1,500 (49.2)	2,509 (51.3)	14,246 (60.6)
Marital status	*p* = 0.007	*p* = 0.245	*p* = 0.006	–
Single	(27.5)	866 (28.1)	1,346 (27.1)	6,925 (29.1)
Married^a^	5,832 (72.5)	2,220 (71.9)	3,612 (72.9)	16,896 (70.9)

^a^Including couples living together

**Table 2:** The distribution of self-reported migraine, MA and MO by lifestyle and socioeconomic factors in women and men

	Women				Men			
	Migraine *n* (%)	MA *n* (%)	MO *n* (%)	Healthy *n* (%)	Migraine *n* (%)	MA *n* (%)	MO *n* (%)	Healthy *n* (%)
School level	*p* < 0.001	*p* < 0.001	*p* < 0.001	–	*p* < 0.001	*p* = 0.351	*p* < 0.001	–
Graduated 12th grade	1,901 (35.2)	774 (35.3)	1,127 (35.1)	4,570 (40.5)	603 (25.5)	225 (28.3)	378 (24.1)	3,577 (30.4)
Graduated 9th grade	2,512 (46.5)	1,035 (47.3)	1,477 (45.9)	4,814 (42.7)	1,139 (48.1)	378 (47.5)	761 (48.5)	5,303 (45.1)
Not Graduated 9th grade	992 (18.4)	381 (17.4)	611 (19.0)	1,891 (16.8)	624 (26.4)	193 (24.3)	431 (27.5)	2,875 (24.5)
Educational level	*p* < 0.001	*p* = 0.193	*p* < 0.001	–	*p* = 0.017	*p* = 0.334	*p* < 0.001	–
Higher education >4 years	301 (6.0)	141 (6.9)	160 (5.3)	858 (8.2)	242 (11.1)	101 (13.8)	141 (9.8)	1,422 (13.1)
Higher education ≤4 years	1,686 (33.4)	664 (32.6)	1,022 (34.0)	3,397 (32.5)	544 (25.0)	197 (26.9)	347 (24.1)	2,636 (24.4)
Vocatinal education	2,089 (41.4)	846 (41.6)	1,243 (41.4)	4,175 (40.0)	1,094 (50.3)	337 (46.0)	757 (52.5)	5,171 (47.8)
No education	965 (19.1)	384 (18.9)	581 (19.3)	2,009 (19.2)	293 (13.5)	97 (13.3)	196 (13.6)	1,585 (14.7)
Employment status	*p* < 0.001	*p* < 0.001	*p* < 0.001	–	*p* = 0.015	*p* = 0.220	*p* = 0.062	–
Employed	3,227 (68.3)	1,287 (66.6)	1,940 (69.6)	6,642 (67.1)	1,593 (72.2)	528 (70.9)	1,065 (72.9)	7,826 (71.3)
Self-employed	215 (4.6)	79 (4.1)	136 (4.9)	415 (4.2)	276 (12.5)	94 (12.6)	182 (12.5)	1,438 (13.1)
Homemaker	265 (5.6)	97 (5.0)	168 (6.0)	559 (5.7)	3 (0.1)	2 (0.3)	1 (0.1)	18 (0.2)
Retired	447 (9.5)	213 (11.0)	234 (8.4)	787 (8.0)	174 (7.9)	64 (8.6)	110 (7.5)	727 (6.6)
Student	421 (8.9)	182 (9.4)	239 (8.6)	1,250 (12.6)	123 (5.6)	44 (5.9)	79 (5.4)	811 (7.4)
Unemployed	148 (3.1)	76 (3.9)	72 (2.6)	244 (2.5)	37 (1.7)	13 (1.7)	24 (1.6)	163 (1.5)
Physical work load	*p* < 0.001	*p* < 0.001	*p* < 0.001	–	*p* = 0.180	*p* = 0.241	*p* = 0.014	–
Mainly seated	1,247 (23.8)	504 (23.9)	743 (23.8)	3,033 (27.7)	634 (27.6)	236 (30.3)	398 (26.3)	3,396 (29.6)
Seated/standing or walking	3,692 (70.6)	1,485 (70.4)	2,207 (70.7)	7,458 (68.1)	1,368 (59.6)	462 (59.4)	906 (59.8)	6,676 (58.1)
Heavy physical work	291 (5.6)	120 (5.7)	171 (5.5)	460 (4.2)	292 (12.7)	80 (10.3)	212 (14.0)	1,416 (12.3)
Recreational physical activity	*p* = 0.001	*p* = 0.026	*p* = 0.011	–	*p* = 0.002	*p* = 0.013	*p* = 0.013	–
No physical exercise	1,005 (19.1)	412 (19.3)	593 (18.9)	1,872 (16.9)	411 (17.5)	124 (15.7)	287 (18.4)	1,931 (16.5)
Light physical exercise	3,476 (65.9)	1,393 (65.2)	2,083 (66.3)	7,396 (66.8)	1,394 (59.4)	489 (62.0)	905 (58.1)	6,660 (56.9)
Heavy physical exercise	794 (15.1)	330 (15.5)	464 (14.8)	1,800 (16.3)	541 (23.1)	176 (22.3)	365 (23.4)	3,112 (26.6)
BMI	*p* < 0.001	*p* = 0.031	*p* < 0.001	–	*P* = 0.276	*p* = 0.793	*p* = 0.166	–
Underweight	214 (4.1)	84 (3.9)	130 (4.1)	401 (3.6)	16 (0.7)	7 (0.9)	9 (0.6)	76 (0.7)
Normal weight	3,440 (65.2)	1,408 (66.1)	2,032 (64.6)	7,559 (68.7)	1,181 (50.7)	401 (51.3)	780 (50.3)	5,871 (50.5)
Overweight	1,161 (22.0)	456 (21.4)	705 (22.4)	2,278 (20.7)	911 (39.1)	306 (39.2)	605 (39.0)	4,695 (40.4)
Obese class I–III	459 (8.7)	182 (8.5)	277 (8.8)	765 (7.0)	223 (9.6)	67 (8.6)	156 (10,1)	978 (8.4)
Smoking status	*p* < 0.001	*p* = 0.001	*p* = 0.011	–	*p* = 0.002	*p* = 0.046	*p* = 0.018	–
Non-smoker	2,354 (43.2)	939 (42.5)	1,415 (43.7)	5,293 (46.6)	916 (38.4)	303 (37.9)	613 (38.7)	5,053 (42.4)
Former smoker	1,185 (21.8)	482 (21.8)	703 (21.7)	2,402 (21.1)	577 (24.2)	197 (24.7)	380 (24.0)	2,734 (22.9)
Current smoker	1,905 (35.0)	786 (35.6)	1,119 (34.6)	3,665 (32.3)	890 (37.3)	299 (37.4)	591 (37.3)	4,129 (34.7)
Alcohol consumption	*p* < 0.001	*p* < 0.001	*p* < 0.001	–	*p* < 0.001	*p* < 0.001	*p* < 0.001	–
Never/seldom	1,823 (33.0)	777 (34.7)	1,046 (31.8)	3,029 (26.4)	377 (15.6)	134 (16.4)	243 (15.1)	1,402 (11.7)
Monthly	1,294 (23.4)	504 (22.5)	790 (24.0)	2,880 (25.1)	441 (18.2)	136 (16.7)	305 (19.0)	1,947 (16.2)
Weekly	2,405 (43.6)	955 (42.7)	1,450 (44.1)	5,586 (48.6)	1,604 (66.2)	545 (66.9)	1,059 (65.9)	8,660 (72.1)
Marital status	*p* = 0.001	*p* = 0.091	*p* = 0.001	–	*p* = 0.955	*p* = 0.959	*p* = 0.915	–
Single	1,515 (27.1)	631 (27.9)	884 (26.5)	3,457 (29.6)	697 (28.5)	235 (28.6)	462 (28.4)	3,468 (28.5)
Married^a^	4,082 (72.9)	1,634 (72.1)	2,448 (73.5)	8,213 (70.4)	1,750 (71.5)	586 (71.4)	1,164 (71.6)	8,683 (71.5)

^a^Including couples living together

**Table 3:** The risk of self-reported migraine, MA and MO by lifestyle and socioeconomic factors in all subjects

	Migraine		MA		MO	
	Crude OR (95% CI)	Adjusted OR (95% CI)^a^	Crude OR (95% CI)	Adjusted OR (95% CI)^a^	Crude OR (95% CI)	Adjusted OR (95% CI)^a^
School level^b^						
Graduated 12th grade	1 (reference)	1 (reference)	1 (reference)	1 (reference)	1 (reference)	1 (reference)
Graduated 9th grade	**1.17 (1.11–1.25)**	**1.08 (1.00–1.16)**	**1.14 (1.05–1.24)**	1.04 (0.94–1.16)	**1.20 (1.12–1.29)**	**1.10 (1.01–1.20)**
Not graduated 9th grade	**1.10 (1.03–1.19)**	0.92 (0.83–1.03)	0.98 (0.88–1.10)	**0.81 (0.69–0.95)**	**1.18 (1.09–1.29)**	1.00 (0.88–1.14)
Educational level						
Higher education >4 years	1 (reference)	1 (reference)	1 (reference)	1 (reference)	1 (reference)	1 (reference)
Higher education ≤4 years	**1.55 (1.40–1.73)**	**1.35 (1.19–1.52)**	**1.35 (1.16–1.56)**	**1.22 (1.02–1.46)**	**1.72 (1.50–1.97)**	**1.44 (1.24–1.69)**
Vocatinal education	**1.43 (1.29–1.58)**	**1.22 (1.07–1.38)**	**1.19 (1.03–1.38)**	1.07 (0.89–1.29)	**1.62 (1.42–1.85)**	**1.33 (1.13–1.56)**
No education	**1.47 (1.31–1.65)**	**1.20 (1.04–1.39)**	**1.26 (1.07–1.48)**	1.08 (0.87–1.34)	**1.64 (1.42–1.89)**	**1.30 (1.08–1.56)**
Employment status						
Employed	1 (reference)	1 (reference)	1 (reference)	1 (reference)	1 (reference)	1 (reference)
Self-employed	**0.80 (0.72–0.88)**	0.90 (0.79–1.02)	**0.74 (0.63–0.88)**	0.84 (0.69–1.02)	**0.83 (0.73–0.94)**	0.93 (0.80–1.08)
Homemaker	**1.39 (1.20–1.62)**	1.17 (0.96–1.41)	**1.37 (1.10–1.70)**	1.15 (0.87–1.51)	**1.41 (1.18–1.68)**	1.18 (0.94–1.48)
Retired	**1.23 (1.12–1.36)**	**1.18 (1.03–1.36)**	**1.46 (1.27–1.67)**	**1.39 (1.15–1.69)**	1.09 (0.97–1.24)	1.05 (0.88–1.25)
Student	**0.79 (0.72–0.88)**	**0.86 (0.75–0.99)**	0.87 (0.76–1.01)	0.99 (0.82–1.20)	**0.74 (0.66–0.84)**	**0.78 (0.66–0.93)**
Unemployed	**1.36 (1.14–1.63)**	**1.24 (1.01–1.53)**	**1.74 (1.38–2.20)**	**1.61 (1.22–2.12)**	1.14 (0.91–1.42)	1.01 (0.77–1.33)
Physical work load						
Mainly seated	1 (reference)	1 (reference)	1 (reference)	1 (reference)	1 (reference)	1 (reference)
Seated/standing or walking	**1.22 (1.15–1.30)**	1.07 (0.99–1.15)	**1.20 (1.09–1.31)**	1.09 (0.97–1.22)	**1.24 (1.15–1.34)**	1.06 (0.97–1.16)
Heavy physical work	1.06 (0.96–1.18)	1.00 (0.87–1.15)	0.93 (0.79–1.09)	0.94 (0.76–1.16)	**1.15 (1.01–1.31)**	1.03 (0.88–1.21)
Recreational physical activity						
No physical exercise	1 (reference)	1 (reference)	1 (reference)	1 (reference)	1 (reference)	1 (reference)
Light physical exercise	**0.93 (0.87–1.00)**	**0.92 (0.84–1.00)**	0.95 (0.86–1.05)	0.96 (0.85–1.09)	0.92 (0.85–1.00)	**0.90 (0.81–0.99)**
Heavy physical exercise	**0.73 (0.67–0.80)**	**0.78 (0.70–0.87)**	**0.73 (0.64–0.83)**	**0.79 (0.67–0.93)**	**0.73 (0.66–0.81)**	**0.78 (0.69–0.89)**
BMI						
Underweight	**1.40 (1.19–1.65)**	**1.25 (1.03–1.53)**	**1.42 (1.13–1.78)**	**1.41 (1.08–1.85)**	**1.39 (1.15–1.69)**	1.14 (0.89–1.47)
Normal weight	1 (reference)	1 (reference)	1 (reference)	1 (reference)	1 (reference)	1 (reference)
Overweight	**0.86 (0.81–0.92)**	**0.81 (0.75–0.87)**	**0.81 (0.74–0.89)**	**0.77 (0.69–0.86)**	**0.90 (0.84–0.96)**	**0.83 (0.76–0.91)**
Obese class I–III	**1.14 (1.04–1.25)**	0.98 (0.87–1.10)	1.06 (0.92–1.22)	0.92 (0.77–1.10)	**1.19 (1.06–1.33)**	1.02 (0.88–1.17)
Smoking status						
Non-smoker	1 (reference)	1 (reference)	1 (reference)	1 (reference)	1 (reference)	1 (reference)
Former smoker	**1.09 (1.02–1.16)**	**1.10 (1.02–1.20)**	1.10 (1.00–1.22)	1.12 (0.99–1.27)	1.08 (0.99–1.17)	1.09 (0.99–1.21)
Current smoker	**1.14 (1.07–1.20)**	**1.10 (1.02–1.19)**	**1.16 (1.06–1.27)**	1.11 (0.99–1.24)	**1.12 (1.04–1.20)**	**1.10 (1.00–1.20)**
Alcohol consumption						
Never/seldom	1 (reference)	1 (reference)	1 (reference)	1 (reference)	1 (reference)	1 (reference)
Monthly	**0.72 (0.67–0.78)**	**0.73 (0.67–0.80)**	**0.65 (0.58–0.72)**	**0.66 (0.58–0.76)**	**0.78 (0.71–0.85)**	**0.78 (0.70–0.87)**
Weekly	**0.57 (0.53–0.60)**	**0.55 (0.51–0.60)**	**0.51 (0.47–0.56)**	**0.51 (0.45–0.57)**	**0.61 (0.56–0.65)**	**0.59 (0.53–0.65)**
Marital status						
Single	1 (reference)	1 (reference)	1 (reference)	1 (reference)	1 (reference)	1 (reference)
Married^c^	**1.08 (1.02–1.14)**	**1.08 (1.00–1.17)**	1.05 (0.97–1.14)	1.10 (0.99–1.23)	**1.10 (1.03–1.18)**	1.07 (0.97–1.17)

All significant values are marked with bold text

^a^Odds ratio adjusted for all other determinants and age

^b^Adjusted for all other determinants except educational level

^c^Including couples living together

**Table 4:** Risk of self reported migraine, MA and MO by lifestyle and socioeconomic factors in women and men

	Migraine		MA		MO	
	Crude OR (95% CI)	Adjusted OR (95% CI)^a^	Crude OR (95% CI)	Adjusted OR (95% CI)^a^	Crude OR (95% CI)	Adjusted OR (95% CI)^a^
*Women*						
School level^b^						
Graduated 12th grade	1 (reference)	1 (reference)	1 (reference)	1 (reference)	1 (reference)	1 (reference)
Graduated 9th grade	**1.25 (1.17–1.35)**	**1.11 (1.02–1.22)**	**1.27 (1.15–1.41)**	1.10 (0.96–1.25)	**1.24 (1.14–1.36)**	**1.12 (1.01–1.25)**
Not graduated 9th grade	**1.26 (1.15–1.39)**	1.03 (0.89–1.20)	**1.19 (1.04–1.36)**	0.97 (0.79–1.20)	**1.31 (1.17–1.47)**	1.07 (0.90–1.29)
Educational level						
Higher education >4 years	1 (reference)	1 (reference)	1 (reference)	1 (reference)	1 (reference)	1 (reference)
Higher education ≤4 years	**1.42 (1.23–1.63)**	**1.26 (1.07–1.50)**	1.19 (0.98–1.45)	1.10 (0.87–1.40)	**1.61 (1.34–1.94)**	**1.39 (1.13–1.72)**
Vocatinal education	**1.43 (1.24–1.64)**	1.19 (1.00–1.41)	**1.23 (1.02–1.50)**	1.07 (0.84–1.36)	**1.60 (1.33–1.91)**	**1.28 (1.03–1.60)**
No education	**1.37 (1.18–1.60)**	1.11 (0.92–1.35)	1.16 (0.94–1.43)	0.98 (0.75–1.29)	**1.55 (1.28–1.88)**	1.22 (0.96–1.55)
Occupational status						
Employed	1 (reference)	1 (reference)	1 (reference)	1 (reference)	1 (reference)	1 (reference)
Self-employed	1.07 (0.90–1.26)	1.11 (0.90–1.36)	0.98 (0.77–1.26)	0.94 (0.70–1.28)	1.12 (0.92–1.37)	1.22 (0.96–1.54)
Homemaker	0.98 (0.84–1.14)	0.88 (0.72–1.06)	0.90 (0.72–1.12)	0.81 (0.61–1.08)	1.03 (0.86–1.23)	0.92 (0.73–1.16)
Retired	**1.17 (1.03–1.32)**	1.12 (0.94–1.33)	**1.40 (1.19–1.64)**	**1.33 (1.05–1.68)**	1.02 (0.87–1.19)	0.98 (0.78–1.21)
Student	**0.69 (0.62–0.78)**	0.87 (0.74–1.02)	**0.75 (0.64–0.89)**	0.96 (0.77–1.20)	**0.66 (0.57–0.76)**	**0.81 (0.66–0.98)**
Unemployed	**1.25 (1.01–1.54)**	1.24 (0.97–1.59)	**1.61 (1.23–2.09)**	**1.60 (1.17–2.20)**	1.01 (0.77–1.32)	1.00 (0.73–1.37)
Physical work load						
Mainly seated	1 (reference)	1 (reference)	1 (reference)	1 (reference)	1 (reference)	1 (reference)
Seated/standing or walking	**1.20 (1.12–1.30)**	1.05 (0.96–1.16)	**1.20 (1.07–1.34)**	1.11 (0.97–1.28)	**1.21 (1.10–1.33)**	1.02 (0.91–1.14)
Heavy physical work	**1.54 (1.31–1.81)**	**1.28 (1.04–1.58)**	**1.57 (1.26–1.96)**	**1.36 (1.02–1.81)**	**1.52 (1.25–1.84)**	1.24 (0.96–1.58)
Recreational physical activity						
No physical exercise	1 (reference)	1 (reference)	1 (reference)	1 (reference)	1 (reference)	1 (reference)
Light physical exercise	**0.88 (0.80–0.96)**	0.91 (0.82–1.02)	**0.86 (0.76–0.97)**	0.91 (0.78–1.06)	**0.89 (0.80–0.99)**	0.91 (0.80–1.04)
Heavy physical exercise	**0.82 (0.73–0.92)**	0.92 (0.80–1.06)	**0.83 (0.71–0.98)**	0.94 (0.77–1.14)	**0.81 (0.71–0.93)**	0.90 (0.76–1.07)
BMI						
Underweight	1.17 (0.99–1.39)	1.12 (0.90–1.39)	1.13 (0.88–1.43)	1.20 (0.90–1.60)	1.21 (0.98–1.48)	1.06 (0.81–1.38)
Normal weight	1 (reference)	1 (reference)	1 (reference)	1 (reference)	1 (reference)	1 (reference)
Overweight	**1.12 (1.03–1.22)**	1.03 (0.93–1.14)	1.08 (0.96–1.21)	1.00 (0.86–1.15)	**1.15 (1.04–1.27)**	1.05 (0.93–1.19)
Obese class I–III	**1.32 (1.17–1.49)**	1.13 (0.97–1.33)	**1.28 (1.08–1.52)**	1.07 (0.86–1.33)	**1.35 (1.16–1.56)**	1.18 (0.98–1.42)
Smoking status						
Non-smoker	1 (reference)	1 (reference)	1 (reference)	1 (reference)	1 (reference)	1 (reference)
Former smoker	**1.11 (1.02–1.21)**	**1.11 (1.00–1.24)**	**1.13 (1.00–1.28)**	1.11 (0.96–1.29)	1.10 (0.99–1.21)	1.12 (0.98–1.27)
Current smoker	**1.17 (1.09–1.26)**	**1.15 (1.05–1.26)**	**1.21 (1.09–1.34)**	**1.14 (1.00–1.31)**	**1.14 (1.05–1.25)**	**1.15 (1.03–1.29)**
Alcohol consumption						
Never/seldom	1 (reference)	1 (reference)	1 (reference)	1 (reference)	1 (reference)	1 (reference)
Monthly	**0.75 (0.68–0.82)**	**0.76 (0.68–0.85)**	**0.68 (0.60–0.77)**	**0.72 (0.61–0.84)**	**0.79 (0.72–0.88)**	**0.79 (0.69–0.90)**
Weekly	**0.72 (0.66–0.77)**	**0.68 (0.62–0.75)**	**0.67 (0.60–0.74)**	**0.63 (0.55–0.72)**	**0.75 (0.69–0.82)**	**0.73 (0.64–0.82)**
Marital status						
Single	1 (reference)	1 (reference)	1 (reference)	1 (reference)	1 (reference)	1 (reference)
Married^c^	**1.13 (1.06–1.22)**	1.10 (1.00–1.21)	1.09 (0.99–1.21)	1.11 (0.97–1.27)	**1.17 (1.07–1.27)**	1.09 (0.97–1.22)
*Men*						
School level^b^						
Graduated 12th grade	1 (reference)	1 (reference)	1 (reference)	1 (reference)	1 (reference)	1 (reference)
Graduated 9th grade	**1.27 (1.14–1.42)**	**1.17 (1.03–1.33)**	1.13 (0.96–1.34)	1.12 (0.92–1.38)	**1.36 (1.19–1.55)**	**1.20 (1.02–1.40)**
Not graduated 9th grade	**1.29 (1.14–1.46)**	**1.19 (1.01–1.41)**	1.07 (0.88–1.30)	1.00 (0.76–1.32)	**1.42 (1.23–1.64)**	**1.31 (1.06–1.60)**
Educational level						
Higher education >4 years	1 (reference)	1 (reference)	1 (reference)	1 (reference)	1 (reference)	1 (reference)
Higher education ≤4 years	**1.21 (1.03–1.43)**	1.07 (0.88–1.30)	1.05 (0.82–1.35)	1.00 (0.75–1.35)	**1.33 (1.08–1.63)**	1.12 (0.88–1.42)
Vocatinal education	**1.24 (1.07–1.45)**	1.00 (0.82–1.22)	0.92 (0.73–1.16)	0.83 (0.61–1.13)	**1.48 (1.22–1.78)**	1.12 (0.87–1.44)
No education	1.09 (0.90–1.31)	0.95 (0.75–1.20)	0.86 (0.65–1.15)	0.80 (0.55–1.16)	1.25 (0.99–1.57)	1.05 (0.79–1.41)
Occupational status						
Employed	1 (reference)	1 (reference)	1 (reference)	1 (reference)	1 (reference)	1 (reference)
Self-employed	1.07 (0.90–1.26)	1.00 (0.85–1.19)	0.97 (0.77–1.22)	1.07 (0.82–1.39)	0.93 (0.79–1.10)	0.97 (0.80–1.19)
Homemaker^d^	–	–	–	–	–	–
Retired	**1.17 (1.03–1.32)**	1.19 (0.94–1.50)	1.31 (1.00–1.71)	1.33 (0.93–1.91)	1.11 (0.90–1.37)	1.11 (0.84–1.48)
Student	**0.69 (0.62–0.78)**	**0.74 (0.56–0.97)**	0.80 (0.59–1.10)	0.93 (0.62–1.40)	**0.72 (0.56–0.91)**	**0.63 (0.45–0.89)**
Unemployed	1.12 (0.78–1.60)	0.94 (0.61–1.46)	1.18 (0.67–2.09)	1.10 (0.57–2.11)	1.08 (0.70–1.67)	0.86 (0.50–1.49)
Physical work load						
Mainly seated	1 (reference)	1 (reference)	1 (reference)	1 (reference)	1 (reference)	1 (reference)
Seated/standing or walking	1.10 (0.99–1.22)	1.00 (0.88–1.14)	1.00 (0.85–1.17)	0.93 (0.76–1.14)	1.16 (1.02–1.31)	1.04 (0.89–1.22)
Heavy physical work	1.11 (0.95–1.29)	0.99 (0.81–1.21)	0.81 (0.63–1.06)	0.82 (0.59–1.14)	**1.28 (1.07–1.53)**	1.08 (0.86–1.37)
Recreational physical activity						
No physical exercise	1 (reference)	1 (reference)	1 (reference)	1 (reference)	1 (reference)	1 (reference)
Light physical exercise	0.98 (0.87–1.11)	0.92 (0.80–1.06)	1.14 (0.93–1.40)	1.08 (0.85–1.37)	0.91 (0.79–1.05)	0.85 (0.72–1.00)
Heavy physical exercise	**0.82 (0.71–0.94)**	**0.83 (0.70–0.98)**	0.88 (0.70–1.12)	0.87 (0.66–1.15)	**0.79 (0.67–0.93)**	**0.81 (0.66–0.98)**
BMI						
Underweight	1.05 (0.61–1.80)	1.20 (0.65–2.22)	1.35 (0.62–2.94)	1.83 (0.82–4.10)	0.89 (0.45–1.79)	0.85 (0.36–2.00)
Normal weight	1 (reference)	1 (reference)	1 (reference)	1 (reference)	1 (reference)	1 (reference)
Overweight	0.97 (0.88–1.06)	**0.87 (0.77–0.98)**	0.95 (0.82–1.11)	0.83 (0.69–1.00)	0.97 (0.87–1.09)	0.89 (0.77–1.02)
Obese class I–III	1.13 (0.97–1.33)	1.01 (0.83–1.23)	1.00 (0.77–1.31)	0.96 (0.70–1.32)	1.20 (1.00–1.44)	1.04 (0.83–1.30)
Smoking status						
Non-smoker	1 (reference)	1 (reference)	1 (reference)	1 (reference)	1 (reference)	1 (reference)
Former smoker	**1.16 (1.04–1.31)**	1.13 (0.98–1.30)	1.20 (1.00–1.45)	1.22 (0.97–1.53)	**1.15 (1.00–1.31)**	1.08 (0.91–1.28)
Current smoker	**1.19 (1.08–1.32)**	1.11 (0.98–1.25)	**1.21 (1.02–1.42)**	1.15 (0.94–1.41)	**1.18 (1.05–1.33)**	1.09 (0.94–1.26)
Alcohol consumption						
Never/seldom	1 (reference)	1 (reference)	1 (reference)	1 (reference)	1 (reference)	1 (reference)
Monthly	**0.84 (0.72–0.98)**	0.87 (0.72–1.06)	**0.73 (0.57–0.94)**	0.73 (0.54–1.00)	0.90 (0.75–1.08)	0.95 (0.76–1.19)
Weekly	**0.69 (0.61–0.78)**	**0.67 (0.57–0.78)**	**0.66 (0.54–0.80)**	**0.67 (0.52–0.86)**	**0.71 (0.61–0.82)**	**0.67 (0.55–0.81)**
Marital status						
Single	1 (reference)	1 (reference)	1 (reference)	1 (reference)	1 (reference)	1 (reference)
Married^c^	1.00 (0.91–1.10)	1.00 (0.88–1.13)	1.00 (0.85–1.17)	1.00 (0.82–1.23)	1.01 (0.90–1.13)	1.00 (0.85–1.16)

All significant values are marked with bold text

^a^Odds ratio adjusted for all other determinants and age

^b^Adjusted for all other determinants except educational level

^c^Including couples living together

^d^The number in this category was negligible and excluded from the logistic regression model

After adjustment, increased risk of migraine and its subtypes was found for low level of schooling and education. The ORs were highest for low educational level whereas low school level showed only a slight increased risk in all subjects and in women. School level and educational level were significantly associated with MO but not to MA. In all subjects there was an increased risk of MA but not MO when being unemployed or retired compared to employed. The risk of MO but not MA was decreased in both sexes when studying at a college or a university. There was a significantly increased risk of MA but not MO in women having a heavy physical work load. For male subjects no association with work load was found. The risk of migraine as well as its subtypes was significantly decreased when doing heavy physical exercise not related to work. When stratifying by sex, this was only significant in men with MO whereas no association was found in women. The risk of MA but not MO was increased in underweight subjects. There was a decreased risk of having any kind of migraine when being overweight but not obese. The risk of having migraine was slightly increased when smoking. All subjects with a history of smoking and women who were current smokers showed significant association. No association to smoking was found in men. Alcohol consumption on a weekly or monthly basis was associated with a decreased risk of migraine and its subtypes. In men this was only significant for subjects with weekly consumption. The difference between the migraine subtype groups and healthy subjects regarding marital status was insignificant.

### MA versus MO

Direct comparison of the risks of MA with MO according to lifestyle and socioeconomic factors is presented in Table 5. For most of the determinants the risk of MA and MO did not differ significantly. However, we found a decreased risk of MA compared to MO in subjects with low school and educational level, though the adjustment attenuated this significance. The risk of MA compared to MO was also decreased in subjects consuming alcohol on a weekly basis. When being unemployed or retired, the risk of MA was increased compared to MO. When stratifying by sex, this tendency was only seen in women.

**Table 5:** The risk of migraine with aura versus migraine without aura in all subjects, women, and men

	All subjects		Women		Men	
	Crude OR (95% CI)	Adjusted OR (95% CI)^a^	Crude OR (95% CI)	Adjusted OR (95% CI)^a^	Crude OR (95% CI)	Adjusted OR (95% CI)^a^
School level^b^						
Graduated 12th grade	1 (reference)	1 (reference)	1 (reference)	1 (reference)	1 (reference)	1 (reference)
Graduated 9th grade	0.95 (0.86–1.06)	0.95 (0.83–1.07)	1.02 (0.90–1.15)	0.97 (0.84–1.13)	0.83 (0.68–1.03)	0.95 (0.74–1.22)
Not graduated 9th grade	**0.83 (0.73–0.95)**	**0.81 (0.67–0.98)**	0.91 (0.78–1.06)	0.92 (0.72–1.17)	**0.75 (0.59–0.95)**	0.79 (0.57–1.09)
Educational level						
Higher education >4 years	1 (reference)	1 (reference)	1 (reference)	1 (reference)	1 (reference)	1 (reference)
Higher education ≤4 years	**0.78 (0.65–0.95)**	0.83 (0.66–1.04)	**0.74 (0.58–0.94)**	0.78 (0.59–1.05)	0.79 (0.58–1.08)	0.85 (0.59–1.23)
Vocatinal education	**0.74 (0.61–0.88)**	0.80 (0.63–1.00)	**0.77 (0.61–0.98)**	0.82 (0.61–1.11)	**0.62 (0.47–0.83)**	0.70 (0.48–1.03)
No education	**0.77 (0.63–0.94)**	0.82 (0.63–1.06)	**0.75 (0.58–0.97)**	0.80 (0.57–1.11)	**0.69 (0.49–0.98)**	0.72 (0.46–1.14)
Employment status						
Employed	1 (reference)	1 (reference)	1 (reference)	1 (reference)	1 (reference)	1 (reference)
Self-employed	0.90 (0.74–1.09)	0.90 (0.71–1.13)	0.88 (0.66–1.17)	0.76 (0.54–1.08)	1.04 (0.80–1.37)	1.08 (0.79–1.49)
Homemaker^c^	0.97 (0.75–1.25)	0.98 (0.71–1.36)	0.87 (0.67–1.13)	0.88 (0.63–1.23)	–	–
Retired	**1.33 (1.13–1.58)**	**1.33 (1.05–1.68)**	**1.37 (1.13–1.67)**	**1.35 (1.02–1.79)**	1.17 (0.85–1.63)	1.21 (0.78–1.86)
Student	1.18 (0.98–1.41)	1.26 (1.00–1.60)	1.15 (0.94–1.41)	1.20 (0.91–1.57)	1.12 (0.77–1.65)	1.52 (0.91–2.55)
Unemployed	**1.54 (1.14–2.06)**	**1.58 (1.11–2.25)**	**1.59 (1.14–2.21)**	**1.62 (1.09–2.40)**	1.09 (0.55–2.16)	1.26 (0.55–2.88)
Physical work load						
Mainly seated	1 (reference)	1 (reference)	1 (reference)	1 (reference)	1 (reference)	1 (reference)
Seated/standing or walking	0.96 (0.87–1.08)	1.02 (0.90–1.17)	0.99 (0.87–1.13)	1.10 (0.93–1.29)	0.86 (0.71–1.05)	0.88 (0.69–1.13)
Heavy physical work	**0.81 (0.66–0.98)**	0.91 (0.71–1.16)	1.04 (0.80–1.34)	1.10 (0.79–1.54)	**0.64 (0.47–0.86)**	0.74 (0.50–1.10)
Recreational physical activity						
No physical exercise	1 (reference)	1 (reference)	1 (reference)	1 (reference)	1 (reference)	1 (reference)
Light physical exercise	1.03 (0.92–1.17)	1.07 (0.92–1.24)	0.96 (0.83–1.11)	0.99 (0.83–1.18)	1.25 (0.99–1.59)	1.28 (0.97–1.70)
Heavy physical exercise	1.00 (0.86–1.17)	1.01 (0.84–1.23)	1.02 (0.85–1.24)	1.03 (0.82–1.31)	1.12 (0.85–1.47)	1.09 (0.78–1.52)
BMI						
Underweight	1.02 (0.78–1.33)	1.23 (0.88–1.71)	0.93 (0.70–1.24)	1.12 (0.79–1.59)	1.51 (0.56–4.09)	2.30 (0.76–7.01)
Normal weight	1 (reference)	1 (reference)	1 (reference)	1 (reference)	1 (reference)	1 (reference)
Overweight	0.90 (0.81–1.01)	0.92 (0.81–1.05)	0.93 (0.82–1.07)	0.95 (0.80–1.12)	0.98 (0.82–1.18)	0.94 (0.75–1.18)
Obese class I–III	0.89 (0.76–1.06)	0.89 (0.72–1.10)	0.95 (0.78–1.16)	0.89 (0.69–1.15)	0.84 (0.61–1.14)	0.95 (0.65–1.39)
Smoking status						
Non-smoker	1 (reference)	1 (reference)	1 (reference)	1 (reference)	1 (reference)	1 (reference)
Former smoker	1.02 (0.91–1.15)	1.03 (0.90–1.19)	1.03 (0.90–1.19)	0.99 (0.83–1.18)	1.05 (0.84–1.31)	1.14 (0.87–1.50)
Current smoker	1.04 (0.93–1.15)	1.01 (0.88–1.15)	1.06 (0.94–1.20)	0.98 (0.84–1.15)	1.02 (0.84–1.25)	1.09 (0.85–1.38)
Alcohol consumption						
Never/seldom	1 (reference)	1 (reference)	1 (reference)	1 (reference)	1 (reference)	1 (reference)
Monthly	**0.83 (0.73–0.94)**	0.86 (0.73–1.01)	**0.86 (0.74–0.99)**	0.92 (0.76–1.10)	0.81 (0.60–1.08)	0.77 (0.53–1.11)
Weekly	**0.85 (0.76–0.94)**	**0.86 (0.75–0.99)**	0.89 (0.78–1.00)	0.86 (0.73–1.02)	0.93 (0.74–1.18)	0.99 (0.73–1.34)
Marital status						
Single	1 (reference)	1 (reference)	1 (reference)	1 (reference)	1 (reference)	1 (reference)
Married^d^	0.96 (0.86–1.06)	1.03 (0.90–1.17)	0.94 (0.83–1.05)	1.02 (0.87–1.20)	0.99 (0.82–1.19)	1.02 (0.79–1.30)

All significant values are marked with bold text

^a^Odds ratio adjusted for all other determinants and age

^b^Adjusted for all other determinants except educational level

^c^The number in this category was negligible and excluded from the logistic regression model

^d^Including couples living together

### Men versus women

In direct comparison, significant associations were found between sex and all determinants in migraine subjects, see Table 6. When stratifying by migraine subtypes, smoking and marital status showed no longer significant associations with MA or MO. Our findings showed that among those who had not graduated 9th grade or 12th grade, the risk of migraine was increased in men compared to women. However, the risk was decreased in men compared to women in those with low education. Direct comparison between men and women in employment status showed a decreased risk of migraine in men compared to women when being unemployed or studying. However, the risk was increased in men compared to women when being self-employed. The risk of migraine was increased in men compared to women for those with heavy physical workload, heavy recreational physical exercise or alcohol consumption on a monthly or weekly basis. The risk of migraine was decreased in men compared to women when being underweight, although this was not significant for MA. The risk was, however, increased in men when being overweight or obese.

**Table 6:** The risk of migraine and its subtypes for men versus women

	Migraine		MA		MO	
	Crude OR (95% CI)	Adjusted OR (95% CI)^a^	Crude OR (95% CI)	Adjusted OR (95% CI)^a^	Crude OR (95% CI)	Adjusted OR (95% CI)^a^
School level^b^						
Graduated 12th grade	1 (reference)	1 (reference)	1 (reference)	1 (reference)	1 (reference)	1 (reference)
Graduated 9th grade	**1.43 (1.27–1.60)**	**1.44 (1.24–1.67)**	**1.26 (1.04–1.52)**	**1.35 (1.06–1.72)**	**1.54 (1.33–1.78)**	**1.49 (1.24–1.80)**
Not graduated 9th grade	**1.98 (1.73–2.27)**	**3.08 (2.49–3.81)**	**1.74 (1.39–2.19)**	**2.42 (1.71–3.43)**	**2.10 (1.78–2.49)**	**3.58 (2.73–4.69)**
Educational level						
Higher education >4 years	1 (reference)	1 (reference)	1 (reference)	1 (reference)	1 (reference)	1 (reference)
Higher education ≤4 years	**0.40 (0.33–0.49)**	**0.39 (0.31–0.50)**	**0.41 (0.31–0.56)**	**0.41 (0.28–0.60)**	**0.39 (0.30–0.50)**	**0.38 (0.28–0.52)**
Vocatinal education	**0.65 (0.54–0.78)**	**0.48 (0.38–0.62)**	**0.56 (0.42–0.74)**	**0.43 (0.29–0.65)**	**0.69 (0.54–0.88)**	**0.50 (0.36–0.70)**
No education	**0.38 (0.31–0.47)**	**0.33 (0.25–0.45)**	**0.35 (0.25–0.50)**	**0.31 (0.19–0.51)**	**0.38 (0.29–0.51)**	**0.34 (0.23–0.50)**
Employment status						
Employed	1 (reference)	1 (reference)	1 (reference)	1 (reference)	1 (reference)	1 (reference)
Self-employed	**2.60 (2.16–3.14)**	**2.21 (1.74–2.81)**	**2.90 (2.12–3.98)**	**3.00 (2.00–4.51)**	**2.44 (1.93–3.08)**	**1.85 (1.38–2.49)**
Homemaker^c^	**–**	**–**	**–**	**–**	**–**	–
Retired	**0.79 (0.66–0.95)**	0.86 (0.66–1.13**)**	**0.73 (0.54–0.99)**	0.88 (0.58–1.35)	0.86 (0.68–1.09)	0.89 (0.62–1.26)
Student	**0.59 (0.48–0.73)**	**0.68 (0.50–0.92)**	**0.59 (0.42–0.83)**	0.77 (0.49–1.23)	**0.60 (0.46–0.79)**	**0.64 (0.43–0.95)**
Unemployed	**0.51 (0.35–0.73)**	**0.55 (0.34–0.87)**	**0.42 (0.23–0.76)**	**0.45 (0.22–0.93)**	**0.61 (0.38–0.97)**	0.70 (0.37–1.30)
Physical work load						
Mainly seated	1 (reference)	1 (reference)	1 (reference)	1 (reference)	1 (reference)	1 (reference)
Seated/standing or walking	**0.73 (0.65–0.82)**	**0.79 (0.68–0.92)**	**0.66 (0.55–0.80)**	**0.67 (0.52–0.85)**	**0.77 (0.66–0.89)**	0.87 (0.72–1.06)
Heavy physical work	**1.97 (1.64–2.38)**	**1.64 (1.26–2.13)**	**1.42 (1.03–1.97)**	1.28 (0.82–2.02)	**2.31 (1.83–2.93)**	**1.87 (1.34–2.60)**
Recreational physical activity						
No physical exercise	1 (reference)	1 (reference)	1 (reference)	1 (reference)	1 (reference)	1 (reference)
Light physical exercise	0.98 (0.86–1.12)	0.98 (0.83–1.16)	1.17 (0.93–1.46)	1.16 (0.87–1.56)	0.90 (0.76–1.06)	0.90 (0.73–1.11)
Heavy physical exercise	**1.67 (1.42–1.95)**	**1.75 (1.42–2.15)**	**1.77 (1.35–2.33)**	**1.96 (1.37–2.81)**	**1.63 (1.34–1.98)**	**1.68 (1.30–2.18)**
BMI						
Underweight	**0.22 (0.13–0.36)**	**0.32 (0.18–0.58)**	**0.29 (0.13–0.64)**	0.46 (0.20–1.07)	**0.18 (0.09–0.36)**	**0.22 (0.09–0.53)**
Normal weight	1 (reference)	1 (reference)	1 (reference)	1 (reference)	1 (reference)	1 (reference)
Overweight	**2.29 (2.05–2.55)**	**2.39 (2.07–2.76)**	**2.36 (1.96–2.83)**	**2.21 (1.74–2.80)**	**2.24 (1.95–2.56)**	**2.50 (2.09–3.00)**
Obese class I–III	**1.42 (1.19–1.68)**	**1.74 (1.37–2.20)**	1.29 (0.96–1.75)	**1.57 (1.05–2.34)**	**1.47 (1.19–1.82)**	**1.82 (1.36–2.45)**
Smoking status						
Non-smoker	1 (reference)	1 (reference)	1 (reference)	1 (reference)	1 (reference)	1 (reference)
Former smoker	**1.25 (1.10–1.42)**	1.09 (0.92–1.29)	**1.27 (1.03–1.56)**	1.24 (0.94–1.62)	**1.25 (1.07–1.46)**	1.01 (0.82–1.25)
Current smoker	**1.20 (1.08–1.34)**	**1.17 (1.01–1.36)**	1.18 (0.98–1.42)	1.27 (0.99–1.63)	**1.22 (1.06–1.40)**	1.12 (0.92–1.35)
Alcohol consumption						
Never/seldom	1 (reference)	1 (reference)	1 (reference)	1 (reference)	1 (reference)	1 (reference)
Monthly	**1.65 (1.41–1.92)**	**1.79 (1.46–2.20)**	**1.57 (1.20–2.04)**	**1.54 (1.09–2.18)**	**1.66 (1.37–2.01)**	**1.92 (1.48–2.49)**
Weekly	**3.23 (2.84–3.66)**	**3.39 (2.84–4.04)**	**3.31 (2.68–4.09)**	**3.41 (2.55–4.54)**	**3.14 (2.68–3.69)**	**3.38 (2.71–4.22)**
Marital status						
Single	1 (reference)	1 (reference)	1 (reference)	1 (reference)	1 (reference)	1 (reference)
Married^d^	0.93 (0.84–1.04)	**0.85 (0.73–0.99)**	0.96 (0.81–1.15)	0.81 (0.63–1.04)	0.91 (0.80–1.34)	0.89 (0.73–1.07)

All significant values are marked with bold text

^a^Odds ratio adjusted for all other determinants and age

^b^Adjusted for all other determinants except educational level

^c^The number in this category was negligible and excluded from the logistic regression model

^d^Including couples living together

### Self-rated health

The self-rated health measured by the SF-12 survey showed that migraine subjects compared to healthy subjects scored significantly lower in the physical component (PC) [mean with standard deviation: 51.6 (9.4) vs. 53.7 (7.5); *p* < 0.001] as well as the mental component (MC) [50.6 (9.3) vs. 52.1 (8.1); *p* < 0.001]. The difference on the mean score for migraine, MA and MO compared to healthy subjects was −2.1, −3.5, and −1.3 for the PC and −1.5, −2.5, and −0.9 for the MC. Men scored slightly higher than women in total, however, the difference on the mean score was greater in women than men for the PC and vice versa for the MC.

## Discussion

The present study showed that there was an inverse association between education and migraine. We also found that more subjects with migraine were retired or unemployed. Migraine subjects often had work with some physical demands and they had less recreational physical activities. They smoked more and drank less compared to healthy subjects. More migraine subjects were underweight and less overweight. In the following we shall discuss these associations in relation to the previous literature. We also discuss whether they might be risk factors for migraine or the consequences of migraine.

This study comprised a large non-selective sample, representative of the Danish population. It was possible in this study to differentiate between MA and MO because of the use of validated diagnostic questions [20]. Furthermore, it was possible to subdivide between men and women and to distinguish between several different diagnostic subgroups because of the large sample size. However, the present study also has weaknesses. The validation of the two questions used to identify migraine cases showed that self-reported migraine was only correct in 75% of cases while 25% had answered falsely positive to the diagnostic question about migraine in general. Furthermore, we missed 23.8% of the migraine patients with the questionnaire used [20]. The diagnosis of migraine without aura was reasonably accurate while the diagnosis of migraine with aura contained many false positives, thus, the sensitivity was good but the specificity was low for the MA diagnosis [20]. The lack of diagnostic precision particularly in MA would, however, tend to diminish the differences between migraine cases and normal subjects. The differences observed in the present study are, therefore, minimum figures for associations between migraine and lifestyle and socioeconomic factors. This study did not take burden of headache into account. It is likely that subjects with mild migraine differ from subjects with severe migraine in terms of response to environmental stimuli and choice of lifestyle.

### Schooling and education

The previous findings regarding association between migraine and education are conflicting. Some studies found an inverse association with educational level suggesting a social causation or downward drift due to migraine. High education was associated with a 14–24% reduction in risk of migraine [21]. A relatively small population-based study (*n* = 1,007) of 21- to 30-year-old subjects found that the highest prevalence of any migraine was found in subjects with less than high school education, whereas those with college education had the lowest prevalence. The OR was 2.3 (95% CI 1.4–3.8) for subjects with less than college education compared to those with college education. The twofold increased risk was also found in the two migraine subtypes [5]. These studies were all US population-based, whereas studies outside the US did not find any significant association between migraine and educational level [6, 7, 22]. To our knowledge, only one non-US study found that education and socioeconomic status were associated with migraine [23]. In the present study we found that subjects with migraine had a shorter education compared to healthy subjects. The fact that this tendency was stronger in MO than in MA agrees with previous studies showing that MO attacks are more frequent and severer [24].

It is unclear whether low education in migraine subjects is due to cognitive impairment or an effect of the burden of migraine. A cohort of 3-year-old children followed for 23 years showed that subjects who later developed migraine had lower performances at age 3–13 on tests of verbal ability, but not on other IQ tests. They also performed less well on school examinations at age 15–17 and had not achieved bachelor degrees as frequently as headache-free subjects [25]. In a population-based study of Danish twins aged 45–67 years, lifetime history of migraine or one of its subtypes had little impact on cognitive function [26]. This study conducted individual-based as well as within pair analyses. There was no evidence of link between educational attainment or cognitive skills and migraine in the individual-based analysis. However, there was a tendency toward fewer years of schooling in subjects with migraine and MO in the within pair analysis. Twins are assumed to have an increased risk of prenatal complications compared to singletons, however, twins and singletons are believed not to differ in cognitive skills [27]. Another possibility is that low education may be related to low socioeconomic class, and the association between this and migraine may have many causes, e.g., stress, unhealthy life-style, etc. In conclusion, the lower educational level is likely to be a consequence of migraine.

### Employment status, physical work load and physical activity

Migraine, in its severe forms, is a disabling disorder which can lead to difficulties in maintaining a job or to early retirement. We found that migraine subjects had more work involving “seated/standing with walk”. Higher education is less physically demanding. This can explain why more healthy subjects had work consisting of “mainly seated.” A previous study found that work with high physical demands was not associated with migraine neither for men nor women [7]. However, we detected an increased risk of migraine in women with heavy physical work. Some studies could not detect any associations between migraine and recreational physical activity neither for men nor women [7, 28]. A Swedish study showed, however, that physically inactive subjects had a higher prevalence of self-reported migraine and/or recurrent headache than physically active subjects [29]. We also found an inverse relation between the risk of migraine and the level of physical exercise. One explanation could be that migraine patients avoid physical exertion because of its precipitating effect on migraine [30]. During attacks of migraine, subjects may also be prevented from performance of sports and other activities.

### BMI

Previous studies showed no association between BMI and migraine [21]. However, obesity was associated with increased frequency of attacks and might be a risk factor for migraine progression [31, 32]. We did not find any association between migraine and obesity but there was an increased risk of migraine when being underweight. Little attention has been paid to the health of underweight people, but one study found that among current smokers, those who were underweight had migraine significantly more often than those of normal weight [33]. The severe pain, nausea and other symptoms may adversely affect the appetite resulting in insufficient nutrition and lower BMI. We also found that the risk of migraine was decreased when being overweight.

### Tobacco

Previous studies showed conflicting results. Some found that cigarette smoking did not influence the risk of migraine [34, 35] while others found that the prevalence of migraine was increased among current smokers compared to former smokers and non-smokers aged 20 to 39. It was decreased for subjects 40 years and older, but there was no associations with the amount of cigarettes in any of the age groups [36]. Another study found that among smokers the prevalence increased by 1/3 in students aged 19–26 with a relationship between the number of cigarettes and the development of migraine attack [37]. A cohort study with a 7–15 years follow-up period showed that the relative risk of migraine was higher in smokers [38]. The risk of nicotine dependence was increased both in MA with OR: 1.8 (95% CI 1.0–3.2) and MO with OR: 2.3 (95% CI 1.3–3.8) [5]. We found a slightly but significantly increased risk of 11–15% of migraine in smokers. This may either be due to smoke precipitation of migraine or to the above documented lower level of education and social status in migraine sufferers.

### Alcohol

Migraine subjetcs consume less alcohol than healthy subjects in most studies [22, 36, 39] but not in other studies [21, 34]. One study reported alcohol abuse/dependence to be increased in MA compared to no migraine with OR: 2.1 (95% CI 1.2–3.9) [5]. We found a decreased risk of migraine and its subtypes in persons consuming alcohol on a monthly basis or more frequently. This finding can be explained by the known migraine precipitating properties of alcohol which probably caused an avoidance reaction [35, 39]. That alcohol alternatively should have a prophylactic effect is highly unlikely.

### Marital status

There has been conflicting results whether marital status is associated to migraine [6, 7]. We found no association.

### Self-rated health-related quality of life

Previous studies showed that migraine patients had a lower score on the PC and on the MC in the short-form surveys. A study using the SF-12 found a difference of 4.2 points for the PC and 7.6 points for the MC [40]. A survey using SF-36 showed a reduction in both PC and MC of more than five points in migraine patients compared to controls [41]. The present study found only a minor but significant reduction of the mean score of both components in migraine subjects compared to healthy subjects. This is most likely due to the difference in migraine severity of the study populations.

Adequate treatment is essential to reduce the social burden of migraine as more people with migraine are unemployed or (early) retired. Migraine may also have a negative effect on schooling and education. More awareness should, therefore, be given to children and adolescents with migraine to ensure adequate treatment which may improve their performance in school or college. With regard to health issues, migraineurs tend to smoke more and exercise less. If migraine is well treated it may enable migraineurs to be more physically active; however, if the lack of physical exercise is due to its precipitating effect there would likely be no change. Smoking may provoke migraine attacks and is unhealthy and risk factors for several diseases, therefore, quitting smoking is highly recommended.

## Conclusion

The present study indicates that adequate prevention and treatment of migraine is important to avoid downward social drift. Other factors may trigger or worsen migraine and their prevention can probably reduce the prevalence or severity of migraine. The present study calls in particular for further investigation of possible risk factors which should be considered in strategies leading to migraine prevention.
